# Supplementary figures and images for: Worldwide exploration of the microbiome harbored by the cnidarian model, Exaiptasia pallida (Agassiz in Verrill, 1864) indicates a lack of bacterial association specificity at a lower taxonomic rank
Source: PeerJ. 2017 May 16;5:e3235. doi: 10.7717/peerj.3235 (PMC5436572; doi:10.7717/peerj.3235)

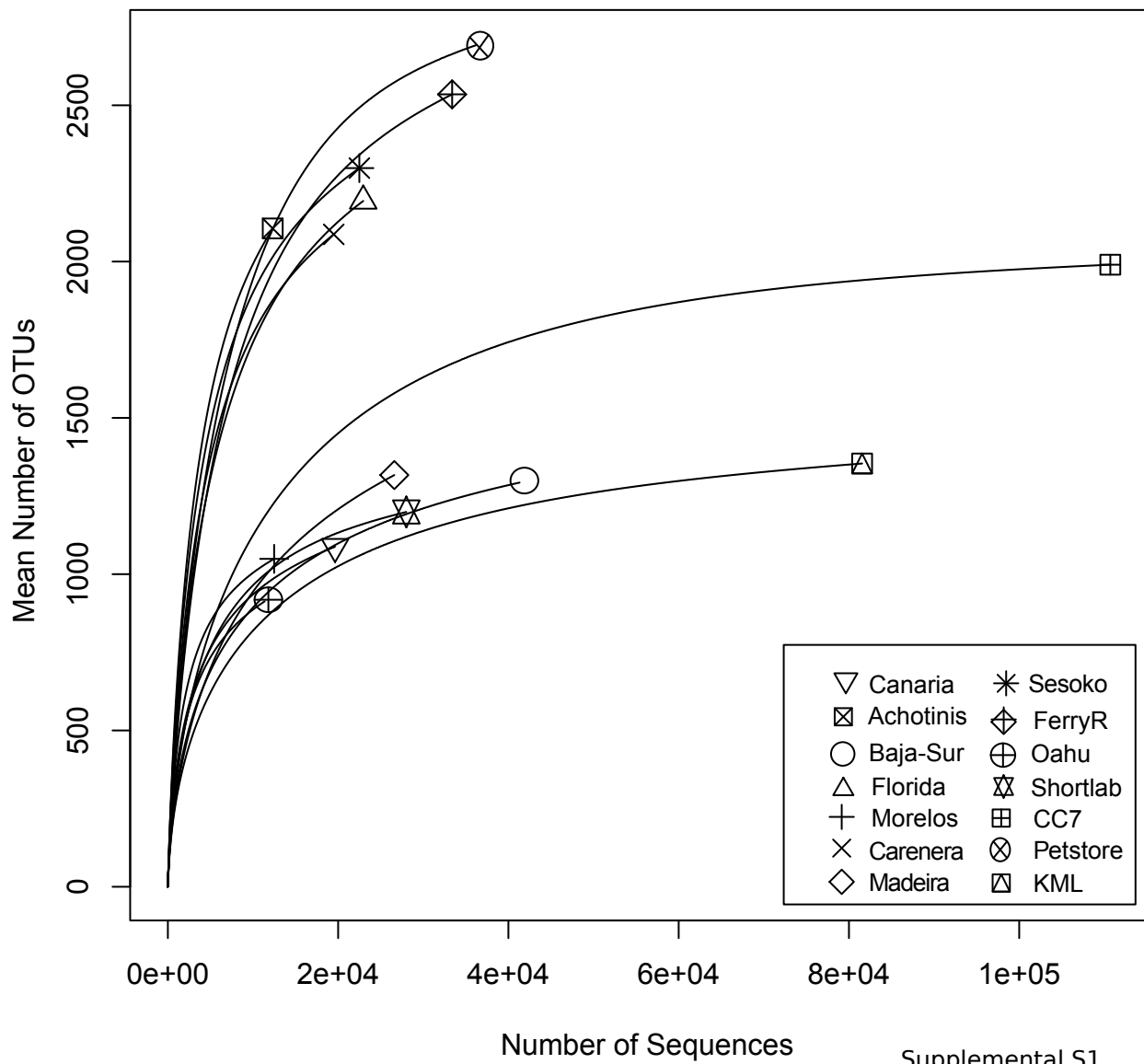

Supplement: Figure S1 [file peerj-05-3235-s001.pdf]

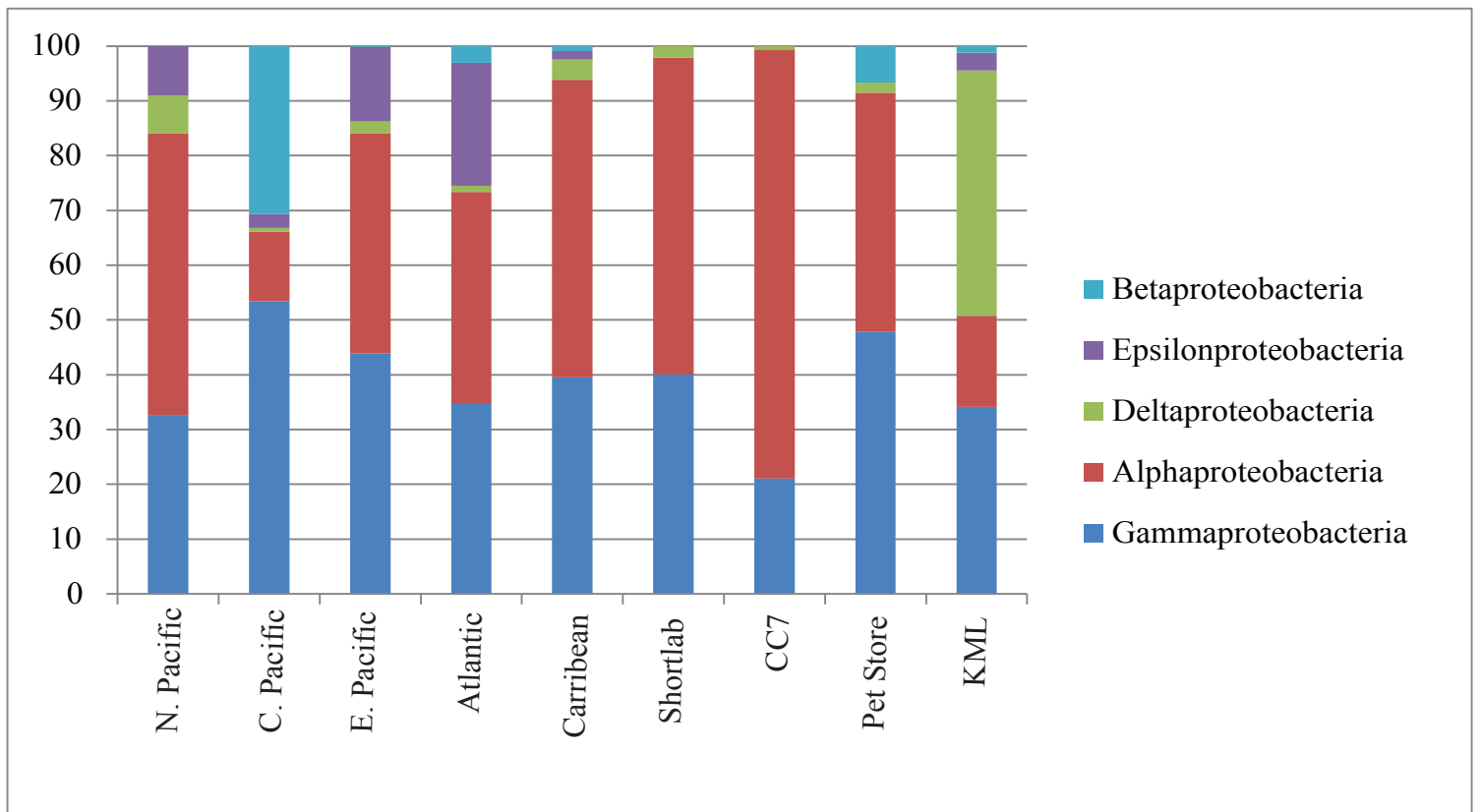

Supplement: Figure S2 [file peerj-05-3235-s002.pdf]

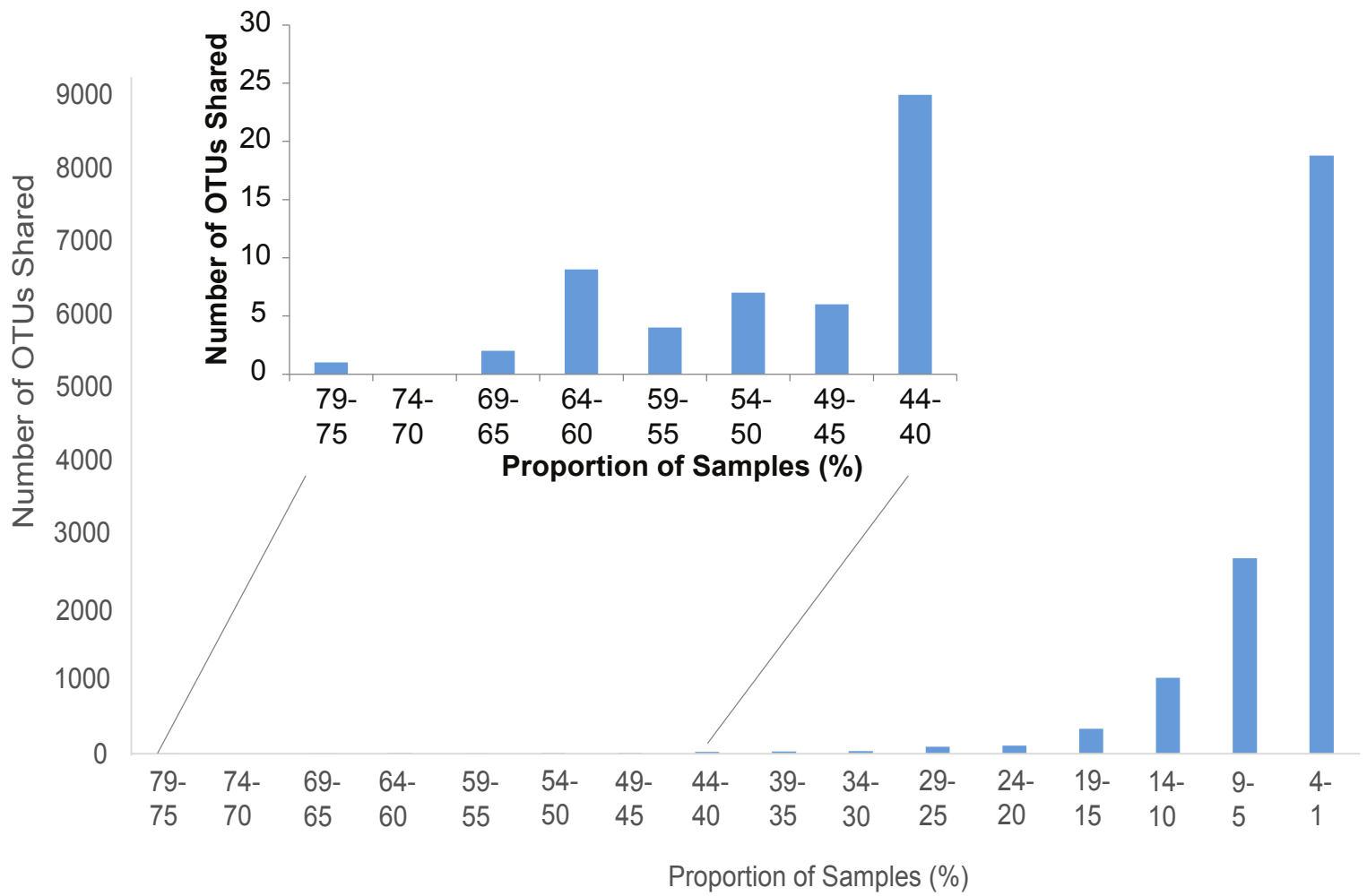

Supplemental S3

Supplement: Figure S3 — Inset shows a close-up section of the main graph displaying the few OTUs shared from 79% to 40% of the analyzed samples. [file peerj-05-3235-s003.pdf]
