# Supplementary material for: Worldwide exploration of the microbiome harbored by the cnidarian model, Exaiptasia pallida (Agassiz in Verrill, 1864) indicates a lack of bacterial association specificity at a lower taxonomic rank
Source: PeerJ. 2017 May 16;5:e3235. doi: 10.7717/peerj.3235 (PMC5436572; doi:10.7717/peerj.3235)
Supplement: Table S1 — Standard deviations are presented along with the averages for both Chao1 and Shannon-Wiener Diversity Indices. [file peerj-05-3235-s004.docx]

Supplemental Table

| **Location ID** | **Location** | **Observed OTUs** | **Expected OTUs (Chao 1)** | **Shannon-Wiener Index** |
| --- | --- | --- | --- | --- |
| Morelos | Puerto Morelos, Mexico (Caribbean) | 417 ± 270 | 593.3 ± 130.7 | 5.39 ± 0.36 |
| Baja-Sur | Pichilingue, Baja California Sur, Mexico  (Pacific) | 490 ± 74 | 517.7 ± 341.5 | 4.56 ± 0.56 |
| Sesoko | Sesoko Island, Okinawa, Japan (Pacific) | 706 ± 571 | 865.7 ± 672 | 5.40 ± 1.04 |
| FerryR | Ferry Reach, Bermuda (Atlantic) | 793 ± 311 | 986 ± 780.5 | 5.17 ± 1.00 |
| Oahu | Oahu Waikiki, Hawaii, USA (Pacific) | 409 ± 296 | 512.2 ± 360.3 | 4.87 ± 0.67 |
| Florida | Florida Keys National Marine Sanctuary, USA (Atlantic) | 684 ± 512 | 878.5 ± 644.2 | 5.15 ± 1.30 |
| Carenera | Carenera Island, Bocas del Toro, Panama (Caribbean) | 649 ± 408 | 819.0 ± 455.3 | 5.29 ± 1.17 |
| Achotines | Achotines lab, Pedasi, Panama (Pacific) | 716 ± 371 | 1014.6 ± 535.2 | 5.73 ± 0.93 |
| Madeira | Madeira Island, Portugal (Atlantic) | 448 ± 186 | 626.2 ± 291.5 | 4.51 ± 0.65 |
| Canaria | Las Palmas Island, Gran Canaria, Spain (Atlantic) | 508 ± 139 | 671.4 ± 291.5 | 5.12 ± 0.51 |
| KML | Outdoor flow-through aquariums at Key Marine Lab, Long Key, Florida, USA | 884 ± 140 | 1018.5 ± 130.5 | 4.82 ± 0.29 |
| Shortlab | Anemones from KML brought to laboratory captivity for 4 Months | 523 ± 209 | 624.0 ± 253.1 | 4.91 ± 0.55 |
| CC7 | Clone CC7 in laboratory captivity for 6 Years | 1358 ± 225 | 1539.8 ± 200.3 | 5.27 ± 0.06 |
| Petstore | Unknown Collection Site | 1671 ± 144 | 1906.3 ± 167.4 | 5.83 ± 0.63 |
