# Supplementary material for: Worldwide exploration of the microbiome harbored by the cnidarian model, Exaiptasia pallida (Agassiz in Verrill, 1864) indicates a lack of bacterial association specificity at a lower taxonomic rank
Source: PeerJ. 2017 May 16;5:e3235. doi: 10.7717/peerj.3235 (PMC5436572; doi:10.7717/peerj.3235)
Supplement: Table S2 [file peerj-05-3235-s005.docx]

**Supplemental table S2**

| **Tube ID** | **Study ID** | **Country** | **Location** | **Latitude** | **Longitude** | **Date Collected** | **Depth** | **Symbiont Type** |
| --- | --- | --- | --- | --- | --- | --- | --- | --- |
| A28 | Morelos | Mexico | Puerto-Morelos | N 20 50' 14'' | W 86 53' 01'' | Unknown | 0-10 m | A4 |
| A29 | Morelos | Mexico | Puerto-Morelos | N 20 50' 14'' | W 86 53' 01'' | Unknown | 0-10 m | A4 |
| A36 | Morelos | Mexico | Puerto-Morelos | N 20 50' 14'' | W 86 53' 01'' | Unknown | 0-10 m | A4 |
| A50 | Sesoko | Japan | Okinawa (Sesoko Island) | N 26 38' 10'' | E 127 51' 60'' | 2/18/2010 | 0-2 m | B1 |
| A52 | Sesoko | Japan | Okinawa (Sesoko Island) | N 26 38' 10'' | E 127 51' 60'' | 2/18/2010 | 0-2 m | B1 |
| A53 | Sesoko | Japan | Okinawa (Sesoko Island) | N 26 38' 10'' | E 127 51' 60'' | 2/18/2010 | 0-2 m | B1 |
| A54 | Sesoko | Japan | Okinawa (Sesoko Island) | N 26 38' 10'' | E 127 51' 60'' | 2/18/2010 | 0-2 m | B1 |
| A56 | FerryR | Bermuda | Ferry Reach | N 32 22' 12 | W 64 61' 47'' | 6/9/2010 | 0-2 m | B1/C1 |
| A59 | FerryR | Bermuda | Ferry Reach | N 32 22' 12 | W 64 61' 47'' | 6/9/2010 | 0-2 m | B1/C1 |
| A63 | FerryR | Bermuda | Ferry Reach | N 32 22' 12 | W 64 61' 47'' | 6/9/2010 | 0-2 m | B1/C1 |
| A64 | FerryR | Bermuda | Ferry Reach | N 32 22' 12 | W 64 61' 47'' | 6/9/2010 | 0-2 m | B1/C1 |
| A76 | Oahu | USA-Hawaii | Oahu, Waikki | N 21 16' 40'' | W 157 50' 01'' | 7/12/2010 | 0-2 m | B1 |
| A79 | Oahu | USA-Hawaii | Oahu, Waikki | N 21 16' 40'' | W 157 50' 01'' | 7/13/2010 | 0-2 m | B1 |
| A80 | Oahu | USA-Hawaii | Oahu, Waikki | N 21 16' 40'' | W 157 50' 01'' | 7/14/2010 | 0-2 m | B1 |
| A116 | Baja-Sur | Mexico-Baja | Baja California Sur (Pichilingue) | N 24 16' 17'' | W 110 19' 34'' | 8/10/2010 | 0-2 m | B1 |
| A118 | Baja-Sur | Mexico-Baja | Baja California Sur (Pichilingue) | N 24 16' 17'' | W 110 19' 34'' | 8/10/2010 | 0-2 m | B1 |
| A120 | Baja-Sur | Mexico-Baja | Baja California Sur (Pichilingue) | N 24 16' 17'' | W 110 19' 34'' | 8/10/2010 | 0-2 m | B1 |
| A125 | Baja-Sur | Mexico-Baja | Baja California Sur (Pichilingue) | N 24 16' 17'' | W 110 19' 34'' | 8/10/2010 | 0-2 m | B1 |
| A173 | Florida | USA-Florida | Florida Keys National Marine Sanctuary | N 25 03’ 36.78” | W 80 25’ 22.92” | 3/10/2011 | 0-3 m | A4/B2 |
| A175 | Florida | USA-Florida | Florida Keys National Marine Sanctuary | N 25 03’ 36.78” | W 80 25’ 22.92” | 3/11/2011 | 0-3 m | A4 |
| A186 | Florida | USA-Florida | Florida Keys National Marine Sanctuary | N 25 03’ 36.78” | W 80 25’ 22.92” | 3/10/2011 | 0-3 m | A4 |
| A189 | Florida | USA-Florida | Florida Keys National Marine Sanctuary | N 24 48’ 11.22” | W 80 48’ 36.84” | 3/14/2011 | 0-3 m | B2 |
| A316 | Carenera | Panama-Bocas | Bocas del Toro/Carenera Island | N 9 20’ 50.34” | W 82 15’ 18.67” | 8/24/2012 | 0-3 m | B1 |
| A317 | Carenera | Panama-Bocas | Bocas del Toro/Carenera Island | N 9 20’ 50.34” | W 82 15’ 18.67” | 8/24/2012 | 0-2 m | B1 |
| A319 | Carenera | Panama-Bocas | Bocas del Toro/Carenera Island | N 9 20’ 50.34” | W 82 15’ 18.67” | 8/24/2012 | 0-2 m | B1 |
| A320 | Carenera | Panama-Bocas | Bocas del Toro/Carenera Island | N 9 20’ 50.34” | W 82 15’ 18.67” | 8/24/2012 | 0-2 m | B1 |
| A513 | Achotines | Panama-Achotines | Achotines lab - Pedasi | N 7 25’ 50.46” | W 80 11’ 36.24” | 9/4/2012 | 0-2 m | B1 |
| A518 | Achotines | Panama-Achotines | Achotines lab – Pedasi | N 7 25’ 50.46” | W 80 11’ 36.24” | 9/4/2012 | 0-2 m | B1 |
| A519 | Achotines | Panama-Achotines | Achotines lab – Pedasi | N 7 25’ 50.46” | W 80 11’ 36.24” | 9/4/2012 | 0-2 m | B1 |
| A520 | Achotines | Panama-Achotines | Achotines lab - Pedasi | N 7 25’ 50.46” | W 80 11’ 36.24” | 9/4/2012 | 0-2 m | B1 |
| A408 | Madeira | Portugal | Madeira, Machico | N 32 42' 52.84'' | W 16 45' 47.85'' | 9/22/2011 | 0-5 m | B1 |
| A409 | Madeira | Portugal | Madeira, Machico | N 32 42' 52.84'' | W 16 45' 47.85'' | 9/21/2011 | 0-5 m | B1 |
| A410 | Madeira | Portugal | Madeira, Machico | N 32 42' 52.84'' | W 16 45' 47.85'' | 9/21/2011 | 0-5 m | B1 |
| A411 | Madeira | Portugal | Madeira, Machico | N 32 42' 52.84'' | W 16 45' 47.85'' | 9/22/2011 | 0-5 m | B1 |
| A427 | Canaria | Spain | Las Palmas Gran Canaria | N 28 08' 53.02'' | W 15 25' 57.01'' | 9/24/2011 | 0-5 m | B1 |
| A429 | Canaria | Spain | Las Palmas Gran Canaria | N 28 08' 53.02'' | W 15 25' 57.01'' | 9/24/2011 | 0-5 m | B1 |
| A430 | Canaria | Spain | Las Palmas Gran Canaria | N 28 08' 53.02'' | W 15 25' 57.01'' | 9/24/2011 | 0-5 m | B1 |
| B1 | CC7 | USA-Florida  Captivity | CC7 | Unknown | Unknown | Unknown | Unknown | A4 |
| B2 | CC7 | USA-Florida  Captivity | CC7 | Unknown | Unknown | Unknown | Unknown | A4 |
| B3 | CC7 | USA-Florida  Captivity | CC7 | Unknown | Unknown | Unknown | Unknown | A4 |
| B4 | Petstore | Unknown  Captivity | Pet Store | Unknown | Unknown | 3/16/14 | Unknown | C3 |
| B5 | Petstore | Unknown  Captivity | Pet Store | Unknown | Unknown | 3/16/14 | Unknown | A4 |
| B7 | KML | USA-Florida  Captivity | Keys Marine Lab | N 24 49.57’ | W 80 48.88’ | 3/16/14 | 0-1 m | A4 |
| B8 | KML | USA-Florida  Captivity | Keys Marine Lab | N 24 49.57’ | W 80 48.88’ | 3/16/14 | 0-1 m | A4 |
| B9 | KML | USA-Florida  Captivity | Keys Marine Lab | N 24 49.57’ | W 80 48.88’ | 3/16/14 | 0-1 m | B2 |
| WA1 | Shortlab | USA-Florida  Captivity | Short Term Lab (From Keys Marine Lab) | N 24 49.57’ | W 80° 48.88’ | 6/10/14 | 0-1 m | A4 |
| WA2 | Shortlab | USA-Florida  Captivity | Short Term Lab (From Keys Marine Lab) | N 24 49.57’ | W 80 48.88’ | 6/10/14 | 0-1 m | A4 |
| WA3 | Shortlab | USA-Florida  Captivity | Short Term Lab (From Keys Marine Lab) | N 24 49.57’ | W 80 48.88’ | 6/10/14 | 0-1 m | A4 |
| WA4 | Shortlab | USA-Florida  Captivity | Short Term Lab (From Keys Marine Lab) | N 24 49.57’ | W 80 48.88’ | 6/10/14 | 0-1 m | A4 |
